# Insights into *Acinetobacter baumannii*: A Review of Microbiological, Virulence, and Resistance Traits in a Threatening Nosocomial Pathogen

**DOI:** 10.3390/antibiotics9030119

**Published:** 2020-03-12

**Authors:** Carole Ayoub Moubareck, Dalal Hammoudi Halat

**Affiliations:** 1 College of Natural and Health Sciences, Zayed University, Dubai P.O. Box 144534, UAE; 2 Department of Pharmaceutical Sciences, School of Pharmacy, Lebanese International University, Beirut, Bekaa Campuses 1103, Lebanon; dalal.hammoudi@liu.edu.lb

**Keywords:** *Acinetobacter baumannii*, multidrug resistance, virulence factors, antibiotics, pathogenesis

## Abstract

Being a multidrug-resistant and an invasive pathogen, *Acinetobacter baumannii* is one of the major causes of nosocomial infections in the current healthcare system. It has been recognized as an agent of pneumonia, septicemia, meningitis, urinary tract and wound infections, and is associated with high mortality. Pathogenesis in *A. baumannii* infections is an outcome of multiple virulence factors, including porins, capsules, and cell wall lipopolysaccharide, enzymes, biofilm production, motility, and iron-acquisition systems, among others. Such virulence factors help the organism to resist stressful environmental conditions and enable development of severe infections. Parallel to increased prevalence of infections caused by *A. baumannii*, challenging and diverse resistance mechanisms in this pathogen are well recognized, with major classes of antibiotics becoming minimally effective. Through a wide array of antibiotic-hydrolyzing enzymes, efflux pump changes, impermeability, and antibiotic target mutations, *A. baumannii* models a unique ability to maintain a multidrug-resistant phenotype, further complicating treatment. Understanding mechanisms behind diseases, virulence, and resistance acquisition are central to infectious disease knowledge about *A. baumannii*. The aims of this review are to highlight infections and disease-producing factors in *A. baumannii* and to touch base on mechanisms of resistance to various antibiotic classes.

## 1. Introduction

Over the last decades, *Acinetobacter baumannii* has globally emerged as a highly troublesome nosocomial pathogen. Its clinical significance has been largely driven by a remarkable ability to acquire or upregulate various resistance determinants, making it one of the most successful multidrug-resistant (MDR) organisms threatening current antibiotic therapy [1]. On top of such fascinating resistance acquisition, *A. baumannii* is endowed with multiple mechanisms of survival under a wide range of environments, potentiating capacity for hospital spread [2]. The attributable mortalities in patients with *A. baumannii* healthcare-associated infections, of which ventilator-associated pneumonia and bloodstream infections are the most common, can range from 5% in general hospital wards to 54% in the intensive care unit (ICU) [3], with increasing reports of community-acquired *A. baumannii* infections [4]. Mounting evidence of extensively drug-resistant (XDR) and pandrug-resistant (PDR) isolates of *A. baumannii* is also accumulating in different countries [5,6,7]. The World Health Organization (WHO) has assigned *A. baumannii* as a critical priority pathogen posing a great threat to human health, and towards which new antibiotics are urgently needed [8]. Such clinical and public health implications underlie the need to further understand and evaluate both disease and antibiotic resistance mechanisms in this pathogen. The aims of the current review are to highlight clinically relevant infections and disease-producing factors in *A. baumannii*, and to revise its mechanisms of resistance to various antibiotic classes.

## 2. Taxonomy and Microbiological Properties of *Acinetobacter baumannii*

The current definition of the genus *Acinetobacter* consists of short, pleomorphic coccobacilli that are Gram-negative, strictly aerobic, catalase-positive, oxidase-negative, nonfermenting, and nonmotile. Their DNA G+C content ranges between 39% to 47%. *Acinetobacter* produces at 37 °C grayish-white, smooth, mucoid colonies on solid media commonly used for diagnostic purposes, like sheep blood agar and tryptic soy agar [2]. 

After its first description at the beginning of the 20th century, this heterogeneous group of bacteria has gone through a remarkable, complex, taxonomic history. Since the 1980s, and in correspondence to wide recognition and emergence of acinetobacters as nosocomial pathogens, refined taxonomy has been constantly updated [4]. Thanks to the work of Bouvet and Grimont [9], an initial landmark classification of acinetobacters was based on DNA–DNA hybridization studies, and distinguished 12 DNA groups or genospecies, some of which were given formal names like *A. baumannii, Acinetobacter calcoaceticus*, *Acinetobacter haemolyticus*, *Acinetobacter johnsonii*, *Acinetobacter junii*, and *Acinetobacter lwoffii*. As of 2019 and according to a review published by Vijayakumar et al., 59 species belong to the genus *Acinetobacter*, where 11 have defined names and 15 have a tentative description [10]. In particular, the *Acinetobacter calcoaceticus–Acinetobacter baumannii* complex (ACB complex) comprises four species: *A. calcoaceticus* (genomic species 1), *A. baumannii* (genomic species 2), *Acinetobacter pittii* (previously genomic species 3), and *Acinetobacter nosocomialis* (previously genomic species 13 TU) that are closely related and difficult to distinguish by phenotypic properties [11]. Recently, two new species, *Acinetobacter seifertii* and *Acinetobacter dijkshoorniae,* were also included within the ACB complex. Hence, the ACB complex collectively includes five *Acinetobacter* species associated with human diseases (*A. baumannii*, *A. pittii*, *A. nosocomialis*, *A. seifertti,* and *A. dijkshoorniae*) and one environmental species (*A. calcoaceticus*), which is commonly isolated from soil and has not been described as a human pathogen [12,13]. 

The identification of *Acinetobacter* to the species level remains complicated and challenging. Phenotypic methods based upon growth temperatures, hemolysis, glucose acidification, and carbon/energy sources [10], in addition to commercial automated systems [14], are proposed. Molecular species identification by DNA–DNA hybridization studies [15], 16S rRNA sequencing [16], and matrix-assisted laser desorption ionization-time of flight mass spectrometry (MALDI-TOF) [17] are increasingly being used. In addition, there is a transition from traditional typing methodology to whole-genome sequencing-based approaches that are proving their utility in epidemiological classification of *Acinetobacter*. Performance of such methods demonstrates superiority to conventional typing and promises useful future incorporation in infection control besides current use in epidemiological research [18,19]. A reliable biomarker for identification of the species *A. baumannii* is the *bla*_OXA-51-like_ gene, chromosomally located in this species and displaying very weak hydrolysis of carbapenems [20]. The utility of this gene for differentiation of *A. baumannii* from ACB complex has been elaborated by Turton and colleagues [21].

*A. baumannii*, *A. pittii*, and *A. nosocomialis* are still difficult to precisely identify in most laboratories and they represent the three most clinically relevant species of *Acinetobacter* that have been implicated in the vast majority of both community-acquired and nosocomial infections [4]. Hence, this review will use *A. baumannii* in the comprehensive sense to refer to these three species. 

## 3. Associated Infections and Clinical Impact of *A. baumannii*

**Figure 1:**
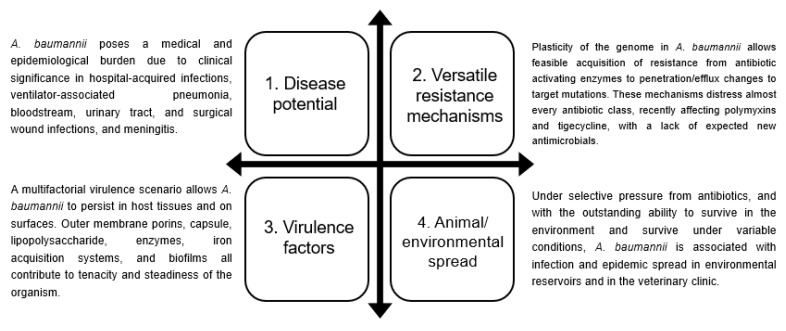
The dynamic microbiological nature of *Acinetobacter baumannii* derives from an interplay between the associated infections, wide arsenal of virulence factors, multidrug-resistant phenotype, and spread in animals and the environment.

As mentioned above, *A. baumanni* has propensity to tolerate stressful environments and multiple classes of antibiotics, making it able to survive and spread as a nosocomial pathogen, particularly in critically ill patients, contributing to increased morbidity and mortality [22]. Previous studies addressing risk factors for acquisition of *A. baumannii* have reported multiple culprits including long ICU stay, previous hospital or ICU stay, previous antimicrobial therapy, mechanical ventilation, use of devices (indwelling catheters, endotracheal tube or nasogastric tube), older age, major or emergent surgery, low birth weight or prematurity, dialysis, and prolonged use of total parenteral nutrition or intravenous lipids [23,24,25,26]. In recent years, *A. baumannii* infections involving respiratory tract, bloodstream, skin and soft tissue, urinary tract, and central nervous system have emerged as highly problematic in healthcare institutions. Figure 1 summarizes *A. baumannii* disease burden, resistance, and endemicity in humans, animals, and the environment. 

### 3.1. Respiratory Infections

Ventilator-associated pneumonia (VAP) caused by MDR *A. baumannii* remains a leading cause of high mortality rate in critically ill patients [27]. *A. baumannii* accounts for 8% to 14% of VAP in the United States and Europe, but this pathogen is associated with higher rates (19% to >50%) in Asia, Latin America, and some Middle Eastern countries [28]. Recent research indicated that incidence of MDR *A. baumannii* transmission was 315.4 cases/1000 ICU patient-days with mortality rate ranging from 52% to 66% [29]. In a seven-year survey of MDR *A. baumannii* infections in a tertiary care center in Lebanon, the most common site of infection among the isolates was the respiratory tract (53.1%), followed by surgical wound (18.8%), blood (15.6%), urine (10.2%), and others (2.3%). The most common colonization site was the respiratory tract (80.8%) followed by skin colonization (12.4%) [30]. In a more recent investigation from a university hospital, rates of MDR, XDR, and PDR *A. baumannii* recovered from VAP cases were 13.3%, 68.3%, and 18.3%, respectively, with female gender and red blood cell transfusion as independent risk factors for mortality [31]. Furthermore, a predominant clonal lineage of *A. baumannii* was found to be associated with VAP across 12 hospital centers in three European countries, and suggests the emergence of a XDR/PDR *A. baumannii* epidemic, with resistant rates close to 100% for carbapenem and 50% for colistin [32]. In a recent meta-analysis involving 29 countries, the prevalence of MDR phenotype among *A. baumannii* causing hospital-acquired pneumonia and VAP was close to 80%. Central America, Latin America, and the Caribbean had the highest prevalence, whereas Eastern Asia had the lowest [33]. Also, in a screening analysis for lower respiratory tract specimens of 97 patients infected with *A. baumannii* in Southern Vietnam, 80% of the specimens were carbapenem resistant and 90% were MDR [34].

Although *A. baumannii*-induced VAP appears to have a predilection for vulnerable patients, community-acquired pneumonia (CAP) due to this organism is an increasing cause for concern. It is characterized by a fulminant course, high incidence of bacteremia, and high mortality rate, especially in tropical regions, where it predominantly affects individuals with excess alcohol consumption, diabetes mellitus, smoking, and chronic lung disease [35]. CAP caused by *A. baumannii* is prevalent in different parts of Australia, Oceania, and Asia, including Taiwan, China, and Thailand [36]. A recent Chinese study reported *A. baumannii* CAP caused by a rare sporadic clone of sequence type 880 that carries the plasmid-encoded *bla*_OXA-72_ gene conferring resistance to carbapenems [37]. Accumulating scientific evidence about the role of *A. baumannii* in respiratory tract infections needs to be put in perspective for better surveillance and control.

### 3.2. Bloodstream Infections

*A. baumannii* has become a leading cause of bloodstream infections in health care settings with intravenous catheters or the respiratory tract representing a frequent source of infection. Mortality rate due to bloodstream infections caused by *A. baumannii* is approaching 40% [38]. High incidence of bacteremia due to *A. baumannii* has been reported in cancer patients, where this organism accounted for about 68% of bacteremia cases in a Brazilian study. Unfortunately, such bacteremia had a high mortality among cancer patients, and risk factors associated with infection-related mortality appeared to be more closely linked to management of bacteremia than to special characteristics of the cancer population, such as neutropenia and tumor site infection [39]. In another category of patients, namely those undergoing neurosurgery, about 13% of bloodstream infections over a period of 10 years were attributed to *A. baumannii*, of which 90% were carbapenem resistant [40]. In another study discussing severely burned patients admitted to a burn ICU, *A. baumannii* was the top isolated pathogen from blood for four consecutive years, and except for low resistance profile to polymyxin B and minocycline, the isolates were almost 100% resistant to other antibiotics [41]. In a recent study from a hospital in Northwest Ethiopia, *A. baumannii* accounted for about 9% of nosocomial bloodstream infections, and were 100% resistant to ampicillin and piperacillin, and 33.3% and 44.5% resistant to meropenem and ciprofloxacin, respectively [42]. Moreover, 42% of *A. baumannii* causing bloodstream infections in ICU patients from a Greek university hospital were resistant to colistin, and were directly linked to fulminant septic shock and high mortality [43]. Such wealth of data regarding the upsurge of drug-resistant *A. baumannii* in bloodstream infections, especially in severely ill patients, requires emphasis on surveillance and characterization of antimicrobial resistance patterns, as well as reinforcement of appropriate antibiotic and infection control measures.

### 3.3. Skin and Soft Tissue Infections

*A. baumannii* has been repeatedly isolated from skin and soft tissue in patients with severe burns, wounds, or trauma, for instance, soldiers injured during military operations or victims of natural disasters. A landmark paper by Davis and colleagues [44] reported war wound infection and osteomyelitis caused by MDR *A. baumannii* during the 2003–2005 military operations in Iraq. In another retrospective study of US military service members wounded in Iraq or Afghanistan, *A. baumanniii* was the most commonly isolated pathogen from open tibial fractures [45]. Afghanistan and Iraq remain the two major geographic locations where *A. baumannii* were isolated from wound or soft tissue especially after traumatic injury [36]. In a military medical center in the US, overall *A. baumannii* isolation increased from 4% to 55% over eight years, with wound isolates accounting for 24% of total *A. baumannii* specimens. Moreover, the percentage of MDR *A. baumannii* isolates recovered was higher among combat casualties deployed overseas (52%) than among local patients (20%) [46]. Among military patients from a tertiary care burn center, patients with *A. baumannii* infection had more severe burns and comorbidities, longer lengths of stay, and higher mortality compared to patients without infection [47]. Therefore, and specifically to military personnel with war wounds, *A. baumannii* represents a challenging threat, and should be prevented by combating nonhealing wounds that are likely to develop wound biofilms (described shortly) with prominent antimicrobial resistance [48]. In one study about Gram-negative osteomyelitis in Brazil, *A. baumannii* caused 21% of cases, and 40% of the isolates were carbapenem resistant [49]. Furthermore, in China, and after the Wenchuan earthquake, about 14% of wound infections in trauma patients were caused by *A. baumannii* [50].

### 3.4. Urinary Tract Infections

Although research on *A. baumannii* infections has primarily focused on pneumonia and bloodstream infections, recent claims are that one in five *A. baumannii* strains are isolated from urinary sites [51]. A. *baumannii* occasionally causes urinary tract infections (UTIs), especially with indwelling urinary catheters [4], and was responsible for 1.6% of ICU-acquired UTIs in one study [52]. Balfousias and colleagues reported a PDR strain of *A. baumannii* causing urinary tract infection in a 72-year old patient admitted to a trauma ward of a Greek hospital for femur fracture, and requiring a combination therapy of rifampicin, tigecycline, and vancomycin in their maximum doses to produce negative urine cultures [53]. In a study of characteristics of *A. baumannii* isolated from ICU in 10 hospitals in Korea, 55.6% of the isolates were associated with urinary tract infection. Of these isolates, 19.8% were resistant to imipenem and 25% to meropenem, 13.5% to polymyxin B, and 17.7% to colistin [54]. Moreover, it is unusual for this organism to cause uncomplicated UTI in healthy outpatients [2].

### 3.5. Meningitis

Nosocomial meningitis due to *A. baumannii* remains an increasing threat in intensive care neurosurgery units, with mortality approaching 70%, especially in patients on indwelling ventriculostomy tubes or cerebrospinal fistulae and receiving post-surgical antimicrobial therapy [55]. The largest case series of postneurosurgical *A. baumannii* meningitis published in 2019 revealed that 21% of isolates had XDR phenotype with sensitivity to only colistin and tigecycline. Furthermore, age over 40, presence of external ventricular drain, raised cerebrospinal fluid white blood cell count, and presence of comorbidities (diabetes and hypertension) were risk factors for mortality due to *A. baumannii* in the neurosurgical ICU [56]. In a review of pediatric meningitis cases caused by *A. baumannii* in China, cultures of cerebrospinal fluid yielded MDR, XDR, and PDR *A. baumannii* following neurosurgery, with high mortality rate [57]. In another report from China, and in a university hospital, the prevalence of MDR and XDR *A. baumannii* infection among patients with intracranial infection after a neurosurgical operation was 33.64%. The isolates were 100% resistant to imipenem and meropenem, 98.38% to cefazolin, 100% to ceftazidime, 100% to cefatriaxone, and 98.39% to cefepime, but completely sensitive to polymyxin B, 60.66% to tigecycline, and 49.18% to amikacin. No significant differences in basic clinical data were observed between the two groups [58]. 

## 4. *A. baumannii* Virulence Factors

**Figure 2:**
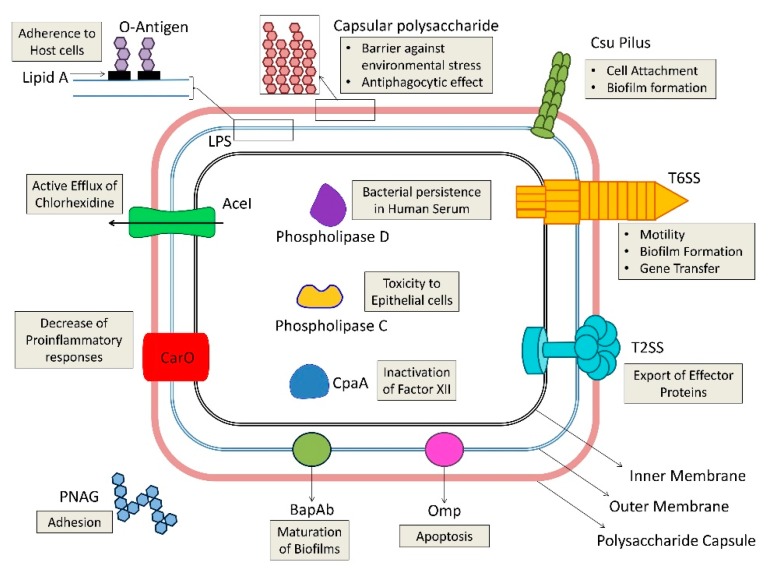
An illustration of virulence determinants possessed by *Acinetobacter baumannii*. The function of each determinant is shown in the adjacent box. AceI = *Acinetobacter* chlorhexidine efflux protein; CpaA = glycan-specific adamalysin-like protease; Csu = chaperon/usher pilus system; LPS = lipopolysaccharide; Omp = outer membrane protein; PNAG = poly-β-1,6-N-acetylglucosamine; T2SS = type II secretion system; T6SS = type VI secretion system.

In recent years, approaches involving genomic, phenotypic, and infection model analyses helped in the identification of virulence factors important for *A. baumannii* pathogenicity [22]. Current consensus supports a multifactorial and combinatorial strategy, with about 16 identified gene islands implicated in virulence, signifying that the organism devotes a considerable portion of its genes to pathogenesis [59]. Systematic studies failed to identify a particular virulence factor responsible for clinical success of *A. baumannii*. This perspective highlights strong adaptive potential and could be a result of adaptation to different human body sites or pathogenic strategies, as known for other bacteria like *Escherichia coli* and *Legionella* [60]. An illustration of virulence determinants identified in *A. baumannii* so far is discussed below and is curtailed in Figure 2.

### 4.1. Outer Membrane Proteins (Porins)

Outer membrane proteins of Gram-negative bacteria generally have a pivotal role in environmental interaction and adaptation, and are key players in virulence [61]. The main outer membrane protein OmpA of *A. baumannii* is involved in cell invasion and apoptosis. This 38kDa protein is vital for small solute penetration. It binds to host cell surface, gets localized in both mitochondria and nuclei, and induces cell death [62]. In a study investigating potential *of A. baumannii* to invade epithelial cells of a murine pneumonia model, prominent lung histopathologic changes (abundance of white blood cells and alveolar damage) were detected in mice having wild-type infection but not in those infected with OmpA^-^ mutants [63]. Besides its transport function as porin, OmpA has the capacity of inducing host cell apoptosis, biofilm formation, dissemination into bloodstream, and interaction with epithelial cells mainly using host fibronectin [64].

Another outer membrane protein of *A. baumannii* is the Omp 33- to 36-kDa protein that acts as a channel for water and whose expression is associated with resistance to carbapenem antibiotics [65]. In one study, this protein was released in immune and connective cells, where it induced apoptosis by blockade of autophagy, enabling intracellular persistence with subsequent development of cytotoxicity [66]. Furthermore, knockout strains of *A. baumannii* with deficient Omp 33–36 had defective growth rate and significantly reduced capability of adherence, invasion, and cytotoxicity, indicating that Omp33–36 plays an important role for fitness and virulence of *A. baumannii* [65].

Like Omp 33–36, CarO plays also a role in carbapenem resistance in *A. baumannii*; it was shown that increased CarO expression delays infiltration of pulmonary neutrophils via attenuation of proinflammatory responses in the trachea and lungs, allowing bacterial proliferation and resulting in severe pneumonia [67]. 

### 4.2. Cell Envelope Factors (LPS and Capsule)

In Gram-negative human pathogens, the cell wall polysaccharide (LPS) is one of the virulence factors involved in multiple steps of disease process. *A. baumannii* LPS is important for resistance to normal human serum and confers a competitive advantage for survival in vivo. It also can elicit a proinflammatory response in animal models [68]. The antigenic O-polysaccharide of the LPS, together with pili, might promote adherence to host cells as a first step of colonization [69].

Beyond the LPS, a major cell structure determinant of *A. baumannii* virulence is the presence of a capsule around bacterial surface. The repetitive, closely packed sugar units of the capsule create a barrier against environmental conditions like dryness and disinfection, and immune system reactions like phagocytosis; it also protects against some antimicrobials [70,71]. Despite differences in capsular sugars of *A. baumannii*, with over 100 variable types, the capsule is perpetually effective for survival of the pathogen during infections and its ability to grow in serum [72].

### 4.3. Enzymes

Phospholipases are known additional virulence factors of *A. baumannii*; these are crucial hydrolytic enzymes and possess a lipolytic activity against phospholipids of human cell membranes. While the enzyme phospholipase D helps *A. baumannii* to persist in human serum as shown in a murine pneumonia model, another enzyme, phospholipase C, is toxic to epithelial cells [73]. 

Data about enzymes in *A. baumannii* continue to culminate; recently, the enzyme CpaA, a glycan-specific adamalysin-like protease, was identified as a virulence factor that inhibits blood coagulation through inactivation of factor XII. As such, CpaA attenuates formation of thrombi in intravascular sites, promoting dissemination capacity of *A. baumannii* [74].

### 4.4. Capsular Polysaccharide Composition and Outer Membrane Resistance to Desiccation and Disinfection

Desiccation resistance, or persistence in dry environments, can allow *A. baumannii* strains to survive up to 100 days, although this period is variable [75]. *A. baumannii* environmental survival is related to presence of capsular polysaccharides surrounding the whole cell and providing defense against the environment [76]. Resistance to dryness has been linked to outer membrane composition; a mutant strain of *A. baumannii* with chemically altered lipo-oligosaccharide was unstable in dryness, suggesting that fluidity of the outer membrane resulting from changes in its lipid structure allows escape of water and nutrients to outside of the cell [77].

*A. baumannii* is shown to actively pump chlorhexidine, an antiseptic used against a wide range of bacteria by disrupting cell membranes. The *Acinetobacter* chlorhexidine efflux protein (AceI) is responsible for such pumping, possibly promoting survival under stressful conditions [78]. On the other hand, ethanol promotes growth and virulence of *A. baumannii* [79]. Ethanol in the serum of alcoholic subjects weakens phagocytosis and hence destruction of the organism [80]. Likewise, excess human consumption of ethanol is a probable predisposing factor for *A. baumannii* community-acquired infections [35].

### 4.5. Biofilm Production and Quorum Sensing

Among all virulence determinants, biofilm formation has become a major pathogenesis feature for *A. baumannii*, making the organism multidrug resistant. By definition, biofilms are microcommunities of microbes enclosed in an extracellular substance, and rendering microbes resistant to stresses including desiccation, immune system clearance, and antibiotics [81]. Biofilms additionally mediate pathogen-host interactions in *A. baumannii*. Most *A. baumannii* have a chaperon/usher pilus system, known as Csu pili, regulated by the BfmRS two-component (TC) system, a network of molecules that influences gene expression and enables building a protective capsule in response to antibiotics. The system also mediates pili formation to facilitate cell attachment [82]. Another TC system, GacSA, can affect Csu gene expression, and therefore may have an indirect influence on the pathogen’s ability to produce biofilms [83]. *A. baumannii* is ‘characterized by production of biofilm-associated proteins (BapAb) similar to Bap protein of *Staphylococcus aureus*, and that have capacity to aggregate for building up biofilm matrix in response to stressful environmental conditions [84]. 

Although most sequenced strains of *A. baumannii* carry a *bap* gene, many seem to have disrupted or truncated *bap* sequences. Nevertheless, Bap-like proteins, BLP1 and BLP2, may be encoded by some strains of *A. baumannii*, and help in maturation of biofilms, like BapAb [85]. Another notable factor that helps *A. baumannii* to produce biofilm is production of the exopolysaccharide poly-β-1,6-N-acetylglucosamine (PNAG), which is produced by many Gram-negative species, and is essential for adhesion and aggregation [86]. 

Many reports suggest that quorum sensing plays a major role in biofilm formation. Quorum sensing is a mode of communication among bacteria to maintain population density, usually by production of signaling molecules known as autoinducers [87]. These are hormone-like compounds, including acyl homoserine lactones (AHLs), that are responsible to regulate motility, biofilm formation, and other characteristics. The quorum sensing cycle in *A. baumannii* includes AbaI inducer as well as its cognate receptor AbaR. AbaI, encoded by the gene *abaI*, is a sensor protein that functions as an autoinducer synthase producing signal AHL molecules, while AbaR functions as a receptor protein, which upon binding to AHLs induces a cascade of reactions. This binding triggers production of more AHLs in a positive feedback loop manner, which results in regulation of biofilm formation [88]. 

### 4.6. Motility

Bacterial motility contributes to the infectious ability and increased virulence of some bacteria [89]. *A. baumannii* lacks flagella and has been long labeled as non-motile [90,91]. However, studies show that this organism can survive during infection and can spread on surfaces during hospital survival by using twitching motility [92]. Such motility consists of extension and retraction movements to push cells in media, by means of type IV pili [93]. In addition to motility, these pili are also responsible for biofilm formation and gene transfer [94]. In *A. baumannii*, a model of *Caenorhabditis elegans* infection showed higher virulence due to motility [95]. Investigations comparing blood isolates of *A. baumannii* to sputum isolates found that the former isolates were more motile, probably indicating a higher survival advantage in blood [96].

In addition to twitching motility, some isolates of *A. baumannii* move on living and non-living surfaces without relying on flagella by another mode of motility called surface-associated motility [97]. This type of motility also requires type IV pili, quorum sensing, lipo-oligosaccahride production, and 1,3-diaminopropane, which mediates signaling needed for impacting surface-associated motility by quorum sensing [90]. 

### 4.7. Micronutrient Acquisition Systems

A major factor contributing to *A. baumannii* persistence as a nosocomial pathogen is its ability to capture host nutrients, including iron, manganese, and zinc, thus adapting to metal-limited environment imposed by the host [98]. The main mechanism used by *A. baumannii* for capturing iron involves five clusters of high-affinity iron-chelating molecules known as siderophores. In addition, the organism possesses transporters and receptors for direct iron uptake such as FecA and FecI, which allow the utilization of heme [99]. Models of iron transporter damage have shown to reduce virulence by decreasing biofilm production and resistance to oxidative stress [100]. 

Moreover, *A. baumannii* relies on a high-capacity zinc scavenging system, consisting of ZnuABC transporter and ZigA GTPase; ZnuABC assures intracellular zinc uptake, while ZigA is responsible for zinc metabolism [101,102]. As such, *A. baumannii* circumvents calprotectin, an immune system protein, that innately complexes zinc, manganese, and divalent metal ions, thus inhibiting bacterial growth. In a murine pneumonia model, *zigA* mutants with depleted zinc availability had less systemic dissemination from the lungs following infection [102]. Although mechanisms employed by *A. baumannii* to overcome manganese limitation are less understood, it is postulated that a transporter belonging to the family of resistance-associated macrophage protein (NRAMP) facilitates manganese accumulation and growth in presence of calprotectin [103]. 

### 4.8. Protein Secretion Systems

In *A. baumannii*, secretion of proteins from cell surface structures allows the pathogen’s interaction with environment and hosts, and hence represents attractive targets for treatment [104,105]. The trimeric autotransporter (Ata) was the first secretion system described in *A. baumannii*. Ata mediates attachment to human matrix components, particularly collagen, and it is also implicated in biofilm formation/maintenance and virulence [104]. *A. baumannii* also uses a type II secretion system (T2SS) to export multiple effector proteins. In this two-step secretion process, the general secretory pathway (Sec) or the Twin-arginine (Tat) system deliver proteins with an N-terminal secretion signal across the pathogen’s inner membrane. Next, the secretion signal is removed, and T2SS machinery secretes folded proteins to outside of the cell [106]. T2SS effectors include CpaA, LipA, and LipH, where LipA and LipH are lipases that are essential for utilization of exogeneous lipids, and CpaA is a metallo-endopeptidase that degrades fibrinogen and factor V in a zinc-dependent mechanism, negatively influencing blood clotting pathways [107]. 

Like many Gram-negative bacteria, *A. baumannii* encodes type VI secretion system (T6SS), a multi-component secretion machine capable of injecting protein toxins into other bacteria in a contact-dependent manner, thus making it important in polymicrobial infections [108]. A single, conserved, genetic locus encodes 13 core structural proteins of this system, while additional, accessory, or regulatory elements may co-exist. Assembled T6SS system contracts to allow release of the effector protein, which typically targets other bacteria without self-intoxication [104]. In *A. baumannii*, T6SS may attack other bacteria, where it produces toxins like nucleases, peptidoglycan hydrolases, or cell-membrane active toxins [109]. Interestingly, clinical isolates with active T6SS are isolated at higher frequencies from immunocompromised patients, suggestive of competitive advantage against concomitant pathogens [110].

### 4.9. Others

Although tangible progress was accomplished in *A. baumannii* pathogenesis studies, emerging areas are still under in-depth investigation. Among these, outer membrane vesicles, long recognized to protect bacteria against host innate immunity, are being studied for their ability to provide mechanisms for secretion of virulence factors in *A. baumannii* [99]. Possibilities also exist that *A. baumannii* may acquire a toxin-like virulence factor like other human pathogens such as *Vibrio cholerae* [111]. Indeed, ongoing research on molecular mechanisms utilized by this pathogen for survival within the host and adaptation to environmental stress could uncover additional important pathogenesis features. 

## 5. Mechanisms of Antibiotic Resistance in *A. baumannii*

The increased antimicrobial resistance in *A. baumannii* and the occurrence of strains resistant to virtually all available drugs is quite alarming [4]. Intrinsically resistant to a number of commonly used antibiotics, such as aminopenicillins, first- and second-generation cephalosporins, *A. baumannii* has a quite notable ability to acquire resistance to numerous other agents and thus swiftly respond to changes in environmental pressure [2]. The current guidelines of treatment of infections by *A. baumannii* are seriously affected by such resistance inclination, rendering options of chemotherapy rather limited [36]. For susceptible organisms, first-line therapy consists of a carbapenem, such as imipenem-cilastatin, meropenem, or doripenem [112], where imipenem was historically considered the gold standard for management of VAP caused by this organism [113]. The clinical cure rates with imipenem for *A. baumannii*-induced VAP range from 57% to 83%. Because isolates susceptible to imipenem may be resistant to meropenem and vice versa, it is advisable to test susceptibility to the specific carbapenem prior to its clinical use [114]. Nevertheless, as explained shortly, carbapenem resistance rates for *A. baumannii* have been rising dramatically worldwide, rendering the antibiotic armamentarium more restricted, and clinical practice is shifted towards alternative agents [36]. Of these, colistin (polymyxin E) and polymyxin B are being used to treat *A. baumannii* VAP, bacteremia and meningitis [113]. A major limitation to colistin is high rates of nephrotoxicity and neurotoxicity, as well as poor penetration into the lung tissue, incumbering its utility [115]. Another alternative agent is minocycline, where successful clinical and microbiologic outcomes were reported for patients with *A. baumannii* VAP as well as skin and soft tissue infections [116]. Tigecycline, an alternative antibiotic for MRD and XDR strains of *A. baumannii*, has been used with variable success rates [117,118]. Despite reasonable in vitro activity of this agent against *A. baumannii*, clinical data remain limited. Specifically, in clinical trials with *A. bauamnnii* VAP and bacteremia, patient outcomes with tigecycline have been inferior to other agents [119]. An interesting alternative to both tetracyclines, minocycline and tigecycline, may be the β-lactamase inhibitor, sulbactam, which has direct antimicrobial activity against *A. bauamnnii*, via its intrinsic affinity for *A. baumannii* penicillin-binding proteins [36]. A major disadvantage of sulbactam is that it is only available in combination with ampicillin in the United States, besides emerging resistance, warranting further investigations into the clinical utility of this agent [120]. 

Combination therapy is frequently used in *A. baumannii* infections as a strategy to increase antibiotic coverage before drug susceptibility testing results are known, to decrease the risk of resistance, and to improve patient outcomes. However, there are no definitive clinical data to support its use for these purposes, and results from human trials are limited [112]. For example, therapy with polymyxin B plus another agent (imipenem, meropenem, rifampin, ampicillin-sulbactam, or others) was associated with a lower mortality rate than with polymyxin B monotherapy [121]. A randomized, multicenter trial done on patients with XDR *A. baumannii* infections treated with colistin and rifampin versus colistin alone, showed superior microbial eradication in patients randomized to the combination arm, but no difference in mortality [122]. Other combination regimens suggest increased efficacy of sulbactam with cefepime, meropenem, imipenem, amikacin, or rifampin [123]. Alternative combinations include colistin-tigecycline and colistin-carbapenem therapy, with the latter perhaps the most supported [36], and have been recommended in several trials [124,125,126]. Furthermore, a recent investigation has highlighted a remarkable synergistic interaction between colistin and vancomycin [127]. 

**Figure 3:**
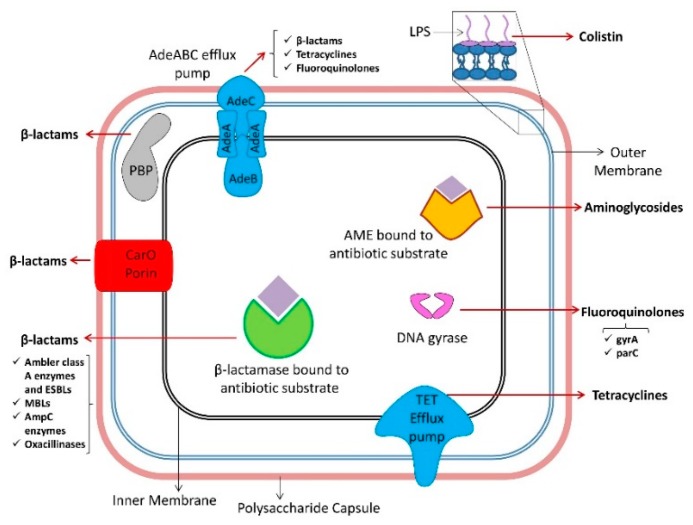
Diagram of various resistance mechanisms of *Acinetobacter baumannii* to antimicrobial agents. Antibiotic modifying enzymes, efflux pumps, porins, drug targets, and the affected antibiotics by each resistance mechanism are shown. AMEs = Aminoglycoside modifying enzymes; AmpC = Ambler class C cephalosporinases; ESBLs = Extended-spectrum β-lactamases; MBLs = Metallo-β-lactamases; LPS = Lipopolysaccharide; PBP = Penicillin binding protein.

A clear consensus regarding the most appropriate combination therapy for resistant *A. baumannii* infections remains to be established, pending more comprehensive clinical studies. Until these become accessible, and until novel antimicrobial entities, bacteriophages, or antimicrobial peptides, which are under thorough scientific investigation, become established, resistance of *A. bauamnnii* to contemporary therapies will escalate. Figure 3 and Table 1 summarize the antibiotic resistance mechanisms in *A. baumannii*; an elaboration of these mechanisms with literature citing them is presented below.

### 5.1. β-lactams

The most prevalent mechanism of resistance of *A. baumannii* to β-lactams is enzymatic hydrolysis by β-lactamases; all of the four Ambler classes of these enzymes have been described in this organism, in addition to nonenzymatic pathways. 

#### 5.1.1. Ambler Class A Enzymes

Class A β-lactamases are serine-dependent enzymes inhibited by clavulanate or tazobactam. They hydrolyze all penicillins and cephalosporins with the exception of cephamycins [128]. CTX-M, GES, PER, SCO, SHV, TEM, and VEB are among identified class A β-lactamases in *A. baumannii* [129,130,131,132]. While some of these, like SCO-1 and TEM-1, are narrow-spectrum, others like CARB-10, CTX-M-2, CTX-M-15, GES-14, PER-1, PER-7, and SHV-5 are extended spectrum β-lactamases (ESBLs) [133,134]. Some GES enzymes with carbapenem-hydrolyzing activity, such as GES-11, have been detected in *A. baumannii* [135,136].

In 2010, Robledo et al. reported, for the first time, Ambler class A *Klebsiella pneumoniae* carbapenemase (KPC)-5 in MDR *A. baumannii* from Puerto Rico [137]. Additional reports described KPC-3 in *A. baumannii* a Portugese University hospital [138], and both KPC-2 and KPC-3 in isolates from Brazil [139]. In a recent meta-analysis about *A. baumannii* in burn injury patients, the prevalence of KPC was over 16% [140]. Carriers of a KPC variants are usually MDR microorganism, and standard medical treatment becomes ineffective with high mortality rates [139]. The true prevalence of KPC and its probable association with other resistance determinants should be investigated carefully, especially with reports of already globally disseminated *A. baumannii* ST2 that carries *bla*_KPC_ [141]. 

#### 5.1.2. Ambler Class B Enzymes

These are zinc-dependent metallo-β-lactamases (MBLs), that strongly hydrolyze all β-lactams, including carbapenems, but not aztreonam [142], and are inhibited by metal chelators like EDTA and dipicolinic acid [128].The most concerning among MBLs is NDM-1, with carbapenem-resistant *A. baumannii* producing NDM-1 being identified in 2011 in China [143]. Additional reports of NDM-1 in *A. baumannii* have accumulated from Tunisia [144], Iran [145], Lebanon [146], and Saudi Arabia [147]. Apart from NDM, VIM [148], GIM [149], SIM [150], and IMP [151,152], including a new allelic variant, IMP-55 [153] were reported.

The majority of acquired MBL genes in *A. baumannii* lie on class 1 integrons, which simultaneously harbor other resistance genes, like aminoglycoside resistance [154]. Such strains are significantly more resistant than strains without integrons, and, clinically, this implies that use of one antibiotic can result in overexpressed resistance to other antibiotics, especially with genetic location of integrons on mobile elements like plasmids or transposons, making them easily transferrable [155].

#### 5.1.3. Ambler Class C Enzymes

Also known as *Acinetobacter*-derived cephalosporinases (ADCs), chromosomally encoded AmpC cephalosporinases are intrinsic to all *A. baumannii* strains [155]. They mediate cephamycin (cefoxitin and cefotetan), cephalosporin, penicillin, and β-lactamase inhibitor combinations resistance, but are not affected by β-lactamase inhibitors like clavulanate and sulbactam [156]. Unlike other Gram-negative bacteria, *A. baumannii* does not induce expression of AmpCs. Overexpression is usually mediated by inserting IS*Aba1* before AmpC genes, enhancing *A. baumannii* resistance to extended-spectrum cephalosporins [157]. Cefepime and carbapenems appear to be stable in the presence of these enzymes [155]. Whole-genome sequencing approaches are allowing detection of new AmpC alleles like AmpC-69, AmpC-70, and AmpC-71 [158]; the new AmpC allelic variant encoded by *bla*_ADC-196_ was also recently identified in a clinical *A. baumannii* isolate from China [159].

#### 5.1.4. Ambler Class D Enzymes

β-lactamases of class D, or the oxacillinases (OXAs), are serine-dependent and commonly hydrolyze oxacillin much faster than benzylpenicillin, hence the name [156]. In various bacteria, over 400 OXA-type enzymes are already known, of which many are carbapenemases [133]. *A. baumannii* isolates harboring plasmid-encoded OXA-23, OXA-24/40, OXA-58, OXA-143, and OXA-235 families have appeared from the 1980s onwards; chromosomal loci encoding some of these enzymes were also identified [20,160,161,162,163]. 

The first isolation of a carbapenem-hydrolyzing class D oxacillinase from *A. baumannii* dates back to 1985, where it was isolated from blood culture of a Scottish patient, and termed ARI-1 [164], which is now referred to as OXA-23 [165]. This enzyme is currently disseminated worldwide [166], and the insertion sequence, IS*Aba1*, in the *bla*_OXA-23_ promoter is associated with overexpression [21]. *A. baumannii* naturally produces chromosomally encoded OXA-51-group carbapenemase at a low level, and acquisition of a strong promoter by IS*Aba1*, analogous to the case with ADCs, upstream of the OXA-51-group gene may lead to elevation of carbapenem MICs [167]. 

It is worth mentioning that OXA-23 enzymes have been identified in many areas of the world in concurrence with other carbapenemases in *A. baumannii* clinical isolates. For instance, OXA-23 co-exists with GES-11 in reports from Lebanon [136] and Kuwait [168], with NDM-1 in reports from India [169], and with OXA-58 in reports from Tunisia [170]. In a study from Thailand, a rare, worrisome case of *A. baumannii* exhibited OXA-23, VIM-2, and NDM-1 [171], highlighting the versatility of carbapenemases that can be harbored simultaneously by this organism.

#### 5.1.5. Nonenzymatic β-lactam Resistance Mechanisms

Apart from β-lactamases, β-lactam resistance in *A. baumannii* is additionally ascribed to nonenzymatic mechanisms, perhaps less well elucidated than enzymatic hydrolysis, but mainly including changes in outer membrane proteins and multidrug efflux pumps [155]. The loss of the 29-kDa outer membrane porin, CarO, was associated with imipenem and meropenem resistance [172]. A recent study from Egypt revealed that IS*Aba1* has been inserted in *carO* gene leading to interruption of its expression [173]. A report about an insertion sequence disrupting the gene encoding the penicillin binding protein PBP6b (also known as dacD) was identified in an endemic carbapenem-resistant clone in a Spanish hospital [174]. Additionally, a collective set of changes involving disruptions in *carO* and *dacD*, as well as carbapenemase production, developed resistance to carbapenems among clinical isolates of *A. baumannii* [175]. The role of other porins like OmpA, Omp33, OprB, Omp25, OprC, OprD, and OmpW is also reported [176].

Multi-drug efflux systems play a major role in β-lactam resistance in *A. baumannii*. The resistance-nodulation-division (RND) family-type pump AdeABC is the best studied and has a broad substrate range including β-lactams, aminoglycosides, erythromycin, chloramphenicol, tetracyclines, fluoroquinolones, and trimethoprim [155]. AdeABC has a three-component structure: Outer membrane protein (*adeC*), multidrug transporter (*adeB*), and membrane fusion protein (*adeA*) [177]. This pump is chromosomally encoded and regulated by a TC system with a sensor kinase (AdeS) and an associated response regulator (AdeR) [178]. Either point mutations or insertion of IS*Aba1* sequence in *adeS* gene leads to overexpression of AdeABC [133]. In a Chinese report, high expression of AdeABC in *A. baumannii* was closely associated with meropenem resistance. The upregulation of *adeA* and *adeB* expression was not due to gene mutations in the regulatory genes*adeS* and *adeR*, suggesting possible involvement of other pathways for efflux pump overexpression [179].

### 5.2. Tetracyclines and Glycylcyclines

Similar to other Gram-negative organisms, tetracycline resistance in *A. baumannii* occurs primarily via energy-dependent efflux pumps, with a lesser extent of resistance attributed to ribosomal protection mechanisms through encoding proteins that shield bacterial ribosomes [180]. Tetracycline efflux pumps of *A. baumannii* fall into two categories: RND pumps, which are nonspecific, constitutive pumps, and Tet efflux pumps with TetA conferring resistance to tetracycline but not minocycline or doxycycline and TetB resulting in resistance to minocycline and tetracycline, tigecycline remaining unaffected [181]. The general structure of RND pumps includes three parts, namely, *AdeA*, *AdeB*, and *AdeC* in *A. baumannii*. These respectively encode membrane fusion, multidrug transporter, and outer membrane pump elements [178]. Gene disruption analysis proved that RND pumps can eject tetracyclines, elevating MICs for tigecyline, minocycline, and tetracycline [182]. Other RND pumps like AdeIJK exist but may play a less profound role on tetracycline efflux and can be synergistic with AdeABC [183]. 

According to studies from many countries, overexpression of the efflux pumps AdeABC and AcrAB-TolC efflux systems was observed in clinical tigecycline-resistant isolates [184,185,186]; this is increasingly affecting utility of this modified tetracycline in treatment. Tigecycline has broader spectrum of activity compared to earlier tetracyclines, has good tissue penetration, and is stable against many tetracycline resistance mechanisms including Tet efflux pumps, as well as against ribosomal protection, such as Tet(O) and Tet(M) [187]. Control of tigecycline usage should be considered to reduce emergence of resistance.

Resistance to minocycline in *A. baumannii* is rare and has been attributed to *tetM*, a ribosomal protection gene [181]. This gene possesses almost 100% homology to *S. aureus* gene, and may represent transfer of resistance mechanisms between the two pathogens [188]. Because *tetM* encodes a GTPase analogous to the elongation factors EF-G and EF-Tu, it can mediate tetracycline release from bacterial ribosome by a GTP-dependent mechanism, through competition with EF-G for an overlapping binding site. By dissociation of tetracycline from its ribosomal binding site, *tetM* enables translation to continue in the presence of tetracycline [189]. Recent evidence shows that tetM is encoded by *A. baumannii* isolated from wastewater treatment plants [190] and animal culture ponds [191], indicating extensive dissemination; the assessment of such resistance pools is critical not only for clinical significance, but also for environmental protection.

### 5.3. Fluoroquinolones

The emergence of resistance to fluoroquinolones in *A. baumannii* results from mutations of the fluoroquinolone target enzymes, DNA gyrase and DNA topoisomerase IV, respectively encoded by the genes *gyrA* and *parC*. [192]. These mutations mainly affect the fluoroquinolone-resistance determining regions (QRDRs) of the target enzymes, with common amino acid substitutions being Ser 83 and Gly 81 within *gyrA*, and Ser 80 and Glu 84 within *parC*. [193]. Such mutations decrease affinity of fluoroquinolones to the enzyme-DNA complex. Clinically significant resistance to fluoroquinolones may be achieved with only a single mutation in *gyrA*; however, double amino acid of both *gyrA* and *parC* produce augmented resistance compared to single ones [194]. There has not been evidence of mutations in *parC* without a concurrent mutation in *gyrA*, which suggests that DNA topisomerase IV could be a complementary target for fluoroquinolones [193]. So far, plasmid-mediated fluoroquinolones resistance genes like *qnrA*, *qnrB*, and *qnrS* have not been identified in epidemiologic studies of *A. baumannii*. 

Moreover, moderate level fluoroquinolone resistance in *A. baumannii* may result from chromosomal efflux pumps [81]. Efflux pump inhibitors can reverse multidrug resistant phenotype of *A. baumannii* [194]. Mutations of a two-step regulator (AdeR) and sensor (AdeS) of the previously mentioned AdeABC efflux pump belonging to RND family of pumps resulted in higher fluoroquinolone efflux [192]. Finally, quinolones are the principle substrates of the efflux pump AbeM, resulting in clinically significant MIC changes for ciprofloxacin and norfloxacin [195]. It is worth mentioning that AbeM is a hydrogen-ion-coupled member of the multidrug and toxic compound extrusion (MATE) family.

### 5.4. Aminoglycosides

Aminoglycoside resistance in *A. baumannii* results from production of aminoglycoside-modifying enzymes (AMEs), which can be categorized into various groups with different chemical actions, including acetyltransferases, adenyltransferases, and phosphotransferases [196]. Such AMEs alter corresponding functional groups on aminoglycosides, weakening the binding capacity of these antibiotics at their ribosomal target sites. AMEs are often found within class 1 integrons and can be located on either plasmids or chromosomes [197]. The action of AMEs is selective, whereby they differently affect various aminoglycoside molecules. For instance, *A. baumannii* phosphotransferases can make many aminoglycosides, including amikacin, inactive. Gentamicin and tobramycin retain activity because of their inability to accept phosphate secondary to a lack of 3′-hydroxyl groups [198]. *A. baumannii* isolates may produce a combination of AMEs, as in a PDR *A. baumannii* strain described in China and carrying four AMEs [199]. Among others, AAC(3)-Ia, ANT(2’)-Ia, and ANT(3″)-Ia are variants of AMEs described in *A. baumannii* [186,200,201].

Another resistance mechanism to aminoglycosides is the production of 16S rRNA methylase genes *armA*, *rmtA*, *rmtB*, *rmtC*, and *rmtD*, which alter target binding site for aminoglycosides within the 30S ribosomal subunit. Unlike AMEs, methylases induce high-level resistance across all clinically useful aminoglycosides, including gentamicin, tobramycin, and amikacin [198]. The *armA* gene is found among other Gram-negative organisms, is plasmid-borne, and lies within a transposon (Tn*1548*) [202].

Although AMEs remain the principle aminoglycoside resistance mechanism in *A. baumannii*, these antibiotics are also subject to efflux pump ejection outside the cell. Gentamicin is subject to effective efflux by AdeABC and AbeM pumps, while these two pumps are less efficient in extruding the more hydrophilic aminoglycosides, amikacin and kanamycin [176,203].

### 5.5. Macrolides

While azithromycin shows variable activity against some *A. baumannii* isolates, this does not really appear significant for clarithromycin and erythromycin [203]. There are only scarce reports in literature on successful treatment of infections caused by *A. baumannii* with macrolides; however, *A. baumannii* with a mutant AbeS small multidrug resistance (SMR) pump exhibits erythromycin and chloramphenicol resistance [204]. Recently, an investigation from Japan [205] proved that the MacA-MacB-TolC tripartite complex transmembrane machine of *A. baumannii* that spans both the inner and outer membranes exists as a unique type of transporter. Macrolides, virulence determinants, peptides, and cell envelop elements appear to be important substrates of this transporter.

### 5.6. Polymyxins

Polymyxin E, also known as colistin, is an old antibiotic belonging to the polymyxin family, first introduced in the 1950s; owing to its harmful effects on renal function, its use was banned by many countries [206]. Nevertheless, the rapid emergence of resistance in *A. baumannii* to multiple antibiotics, including carbapenems, has revived interest in the use of colistin. Currently, resistance to this latter antibiotic in *A. bauamannii* is on the rise [207]. 

Primary colistin resistance mechanisms in *A. baumannii* are chromosomally encoded, and involve (i) phosphoethanolamine (PetN) addition to lipid A of the outer membrane altering its structure, (ii) mutation of genes needed for lipid A synthesis leading to its complete loss, (iii) of outer membrane low expression of proteins needed for outer membrane stability, and (iv) deficient expression of LPS synthesis cofactors [207]. Polymyxins are cationic amphipathic compounds, and initially interact with the negatively charged lipid A component of LPS. Controlled addition of positively charged residues such as PetNt to LPS reduces negative charge on bacterial surface and therefore limits interaction between the polymyxin and the LPS [208]. The expression of PetN transferases is regulated by the concerted action of TS systems [209]. Colistin resistance in *A. baumannii* clinical isolates is associated with alterations in the *pmrCAB* operon. The *pmrC* gene codes for a PetN transferase, and *pmrA* and *pmrB* code for TS system. Mutations in the PmrAB TS system induce overexpression of *pmrC*, leading to modification of lipid A with PetN and colistin resistance [210]. Uniquely, *A. baumannii* strains can also become highly resistant to polymyxins via spontaneous mutations in lipid A biosynthesis such that they produce no LPS or lipid A. If the biosynthetic lipid A genes, *lpxA*, *lpxC*, or *lpxD*, become completely inactive, LPS is lost, elevating colistin MIC due to lack of LPS interaction with this antibiotic [211]. 

Recently, additional genes (*lpsB*, *lptD*, and *vacJ*) were shown to contribute to polymyxins resistance in *A. baumannii*. These genes reduce fluidity and increase osmotic resistance of the outer membrane. In overexpression mutations of these genes, polymyxin resistance in *A. baumannii* occurs [212]. The levels of biotin are essential for susceptibility to polymyxins in *A. baumannii*. Biotin is a co-factor of lipid metabolism, involved in a rate-limiting step in fatty acid synthesis. Accordingly, high levels of biotin promote increased lipid A synthesis, with higher sensitivity to colistin. On the other hand, mutations in genes needed for biotin synthesis reduces effectiveness of colistin [212]. Hood and colleagues showed that removal of a particular locus of *lpsB*, which is involved in biotin biosynthesis can result in colistin resistance in *A. baumannii* [213].

Resistance to colistin in *A. baumannii* was originally chromosomal, which limits its rapid distribution and dissemination [207]. However, the plasmid-borne *mcr-1* gene was identified in *Escherichia coli* of animal, human, and environmental origin from China in 2015 [214]. Subsequently, *mcr-1.2*, *mcr-2*, *mcr-3*, *mcr-4*, and *mcr-5* variants were also identified [215]. Diverse plasmids with *mcr* genes are nowadays described in *Enterobacteriaceae* from many countries. This gene encodes a PetN transferase resulting in polymyxin resistance. Despite that, *mcr* has not been described in *A. baumannii* until recently in reports from Pakistan [206] and Brazil [216], probably highlighting the high tendency for spread and stressing the need to understand the actual status of global colistin resistance in this pathogen.

**Table 1:** Important resistance determinants in *Acinetobacter baumannii* showing possible enzymes/target modifications/permeability lesions.

Antibiotic	Resistance Mechanism	Enzyme/target/ Permeability Defect	Example	Reference
**β-lactams**	β-lactamases	Ambler class A	ESBLs of the families TEM, SHV, CTX-M, PER and VEB	[129,130,131,132]
			SCO-1 (narrow spectrum)	[134]
			Carbapenem-hydrolyzing ESBL of GES-type (GES-11)	[135,136]
			KPC-2	[139]
			KPC-3	[138,139]
			KPC-5	[137]
		Ambler class B	NDM	[143,144,145,146,147]
			VIM	[148]
			GIM	[149]
			SIM	[150]
			IMP	[151,152,153]
		Ambler class C	AmpC-69, AmpC-70, AmpC-71, and ADC-196	[2,157,158]
		Ambler class D	OXA-23-like	[20,136,164,165,167,170]
			OXA24/40-like	[159]
			OXA-51-like	[20,166]
			OXA-58-like	[169]
			OXA-143-like	[161]
			OXA-235-like	[162]
	Permeability lesions	Outer membrane porin downregulation	CarO	[171,172,174]
			OmpA, Omp33, OprB, Omp25, OprC, OprD, and OmpW	[175]
	Efflux pump overactivity	RND pump	AdeABC	[2,176,177]
	Target mutation	PBP	PBP6b (dacD)	[173,174]
**Tertacyclines**	Efflux pump overactivity	RND pump	AdeABC, AdeIJK, and AcrAB-TolC	[179,180,181,182,183,184,185]
		Tet pump	TetA and TetB	[180]
	Ribosomal protection	Tetracycline dissociation from ribosome	Tet(O) and Tet(M)	[180,186,187,188]
**Fluoroquinolones**	Target mutation	DNA gyrase	GyrA	[191,192]
		DNA topoisomerase IV	ParC	[191,192]
	Efflux pump overactivity	RND pump	AdeABC	[81,191]
		MATE family	AbeM	[194]
**Aminoglycosides**	Drug inactivating enzymes	Aminoglycoside modifying enzymes	AAC(3)-Ia	[185]
			AAC(3′)-Ia	[200]
			ANT(2′)-Ia	[200]
			ANT(3″)-Ia	[199]
	Target mutation	16sRNA methylase genes	*armA*, *rmtA*, *rmtB*, *rmtC*, and *rmtD*	[197,201]
	Efflux pump overactivity	RND pumps	AdeABC	[175,202]
**Macrolides**	Efflux pump overactivity	SMR pump	AbeS	[204]
**Polymyxins**	Target mutation	Lipid A modification by PetN transferase	PmrC	[208,209,210]
			MCR-1	[214]
			MCR-4	[215,216]
		Lack of lipid A biosynthesis	LpxA, LpxC, or LpxD	[211]
		Decreased stability of outer membrane	LpsB, LptD, and VacJ	[212]
		Reduced biotin synthesis	LpsB	[207,212,213]

ESBLs = Extended-spectrum β-lactamase; PBP = Penicillin binding protein; RND = resistance-nodulation-division; MATE = multidrug and toxic compound extrusion; SMR = small multidrug resistance; PetN = phosphoethanolamine.

## 6. Conclusions

*A. baumannii* has developed three basic properties to perfectly adapt to current healthcare settings: (i) Ability to colonize skin, mucous membranes, and devices and survive in the hospital environment; (ii) ability to express multiple virulence features; and (iii) extensive resistance to antimicrobial agents through enzymatic modification of antibiotics, target gene mutation, altered outer membrane permeability, and upregulated multidrug efflux pumps. With rapid increase in studies addressing the entire armamentarium of virulence determinants and resistance pathways possessed by this superbug, its complex influences on human health are gradually uncovered. Indeed, thorough investigations will reveal additional knowledge about this staggering pathogen, and the future holds promise for better insights into its machineries through novel research directions.
